# Transcriptional network classifiers

**DOI:** 10.1186/1471-2105-10-S9-S1

**Published:** 2009-09-17

**Authors:** Hsun-Hsien Chang, Marco F Ramoni

**Affiliations:** 1 Childrens' Hospital Informatics Program, Harvard-MIT Division of Health Sciences and Technology, Harvard Medical School, Boston, Massachusetts, USA

## Abstract

**Background:**

Gene interactions play a central role in transcriptional networks. Many studies have performed genome-wide expression analysis to reconstruct regulatory networks to investigate disease processes. Since biological processes are outcomes of regulatory gene interactions, this paper develops a system biology approach to infer function-dependent transcriptional networks modulating phenotypic traits, which serve as a classifier to identify tissue states. Due to gene interactions taken into account in the analysis, we can achieve higher classification accuracy than existing methods.

**Results:**

Our system biology approach is carried out by the Bayesian networks framework. The algorithm consists of two steps: gene filtering by Bayes factor followed by collinearity elimination via network learning. We validate our approach with two clinical data. In the study of lung cancer subtypes discrimination, we obtain a 25-gene classifier from 111 training samples, and the test on 422 independent samples achieves 95% classification accuracy. In the study of thoracic aortic aneurysm (TAA) diagnosis, 61 samples determine a 34-gene classifier, whose diagnosis accuracy on 33 independent samples achieves 82%. The performance comparisons with three other popular methods, PCA/LDA, PAM, and Weighted Voting, confirm that our approach yields superior classification accuracy and a more compact signature.

**Conclusions:**

The system biology approach presented in this paper is able to infer function-dependent transcriptional networks, which in turn can classify biological samples with high accuracy. The validation of our classifier using clinical data demonstrates the promising value of our proposed approach for disease diagnosis.

## Background 

Genome-wide expression analysis has revolutionized disease diagnostic models through the identification of molecular signatures [1], which are selected from high ranked genes determined by statistical measures, such as fold change [2], *t* statistic [3], signal-to-noise ratio [4], or subnetwork scores [5]. Over the last decade, system biology researchers also exploited the comprehensive transcriptional landscape offered by microarrays to identify the transcriptional networks that unravel regulatory gene interactions and explain how diseases progress [6-8]. Although these two analysis approaches seem antithetic, they can be unified to create *transcriptional network classifiers* to enhance disease diagnosis accuracy. We can regard the transcriptional networks underpinning disease development as perturbed by the presence of diseases. The phenotype is treated as a binary perturbation of the overall transcriptional network. To reconstruct the classifier, our task is just to infer from expression profiles the function-dependent transcriptional network that modulates phenotypic traits.

**Figure 1:**
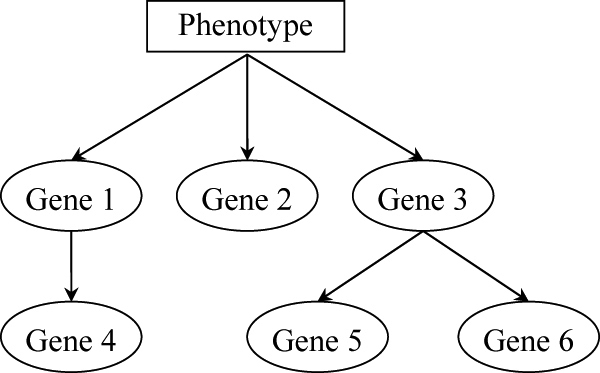
**An example Bayesian network.** A node represents a variable, and a directed arc linking a pair of nodes records the conditional probability of the child (target) node on the parent (source) node. In this network, genes 1, 2, 3 are under the phenotype's Markov blanket, so they form a signature for phenotype classification.

Gene interactions play a central role in transcriptional networks. Abnormal interactions between gene transcripts will give rise to disease incursion [8,9]. To develop transcriptional network classifiers, we consider a system biology approach to capture gene interactions through the measurements of expression collinearity between genes. Our approach is carried out by the Bayesian networks framework, which is a powerful instrument to delineate dependence networks among variables. Bayesian networks have been extensively applied to analyze several types of genomic data, including gene regulation [10], protein-protein interactions [11], single-nucleotide polymorphisms [12] and pedigrees [13]. A Bayesian network is a directed acyclic graph in which nodes represent random variables and arcs define directed dependencies quantified by probability distributions. This study considers a mixed Bayesian network, where the tissue type is represented by a discrete variable and gene expression levels are modelled by continuous, log normal, distributions. Figure 1 illustrates a Bayesian network, where a node represents a gene or a phenotype, and a directed arc linking a pair of nodes records the conditional probability of the child (target) node on the parent (source) node. 

Both the graphical structure of a Bayesian network and the parameters of the conditional probabilities can be learned from the available database. Nevertheless, learning a network is computationally intensive because ideally the dependent relations of all pairs of variables must be evaluated. We circumvent the demanding computations by a two-stage learning process. Our algorithm begins with the use of Bayes factor to select the genes that are functionally dependent on the phenotype, since only function-dependent genes have potential to play a role in tissue discrimination. Then, we explore the detailed dependencies between the selected genes to reconstruct a transcriptional network. After the transcriptional network is learned, it can be exploited for tissue classification, again formulated in the Bayesian networks framework. In the learned network, the phenotype's Markov blanket is the set of nodes composed of the phenotype's parents, its children, and its children's parents. Given the genes under the Markov blanket, the phenotype is independent of the genes not covered by the Markov blanket. Hence, only the genes under the Markov blanket contribute to phenotype classification, and they assemble a signature. With reference to Figure 1, genes 1, 2, 3 are those under the phenotype's Markov blanket, consisting of a signature for tissue classification. 

## Results

We validate our approach by two clinical studies: discrimination of lung cancer subtypes and diagnosis of thoracic aortic aneurysm.

### Discrimination of lung cancer subtypes

**Figure 2:**
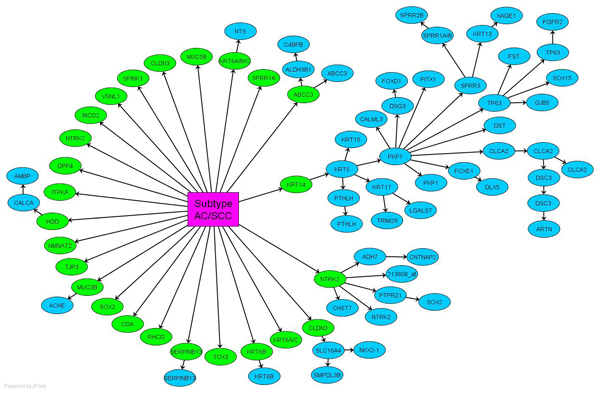
**The functional dependence network for lung cancer subtypes characterization.** There are 77 genes dependent on the lung cancer subtypes and they are selected to build up this network, where 25 genes (in green) are under the phenotype's Markov blanket to assemble a signature.

Lung adenocarcinoma (AC) and squamous cell carcinoma (SCC) are the most common subtypes of lung cancer. They are heterogeneous in many clinical aspects, such as responses to chemotherapy [14], tendency to metastases  [15,16], and mortality rates [17,18]. Unfortunately, the current gold standard is histology which is subjective [19] and may fail when tumors are small [20] or when patients suffer from multiple types of primary lung carcinomas [21]. Gene expression profiling will avoid these problems and perform automatic discrimination of lung cancer subtypes. The classifier is trained by a Duke University data [22], which is available on Gene Expression Omnibus with accession number GSE3141, in a total of 58 ACs and 53 SCCs. The lung specimens are assayed by Affymetrix HG-U133A. Figure 2 shows the function-dependent transcriptional network inferred from the data. Of the 22,283 gene probes in the microarray, seventy seven probes are dependent, directly or indirectly, on the carcinoma subtypes. Of these 77 genes, 25 are under the phenotype Markov blanket, so they *per se* assemble a signature. Enrichment study shows that there are 23 unique genes in this signature, summarized in Table 1.

**Table 1:** The signature of 25 genes for characterizing lung cancer subtypes. Enrichment shows that there are 23 unique genes in the signature.

**Gene symbol**	**Gene title**	**Pathway**
*ABCC3*	ATP-binding cassette, sub-family C (CFTR/MRP), member 3	ABC transporters
*BICD2*	bicaudal D homolog 2 (Drosophila)	
*CDA*	cytidine deaminase	Pyrimidine metabolism, Drug metabolism
*CLDN3*	claudin 3	Cell adhesion molecules, Tight junction, Leukocyte transendothelial migration
*DPP4*	dipeptidyl-peptidase 4	
*HGD*	homogentisate 1,2-dioxygenase (homogentisate oxidase)	Tyrosine metabolism, Styrene degradation
*ITPKA*	inositol 1,4,5-trisphosphate 3-kinase A	Inositol phosphate metabolism, Calcium signaling pathway, Phosphatidylinositol signaling system
*KRT14*	keratin 14 (epidermolysis bullosa simplex, Dowling-Meara, Koebner)	Cell communication
*KRT6A, KRT6B, KRT6C*	keratin 6A, keratin 6B, keratin 6C,	Cell communication
*MUC3B*	mucin 3B, cell surface associated	
*MUC5B*	mucin 5B, oligomeric mucus/gel-forming	
*NMNAT2*	nicotinamide nucleotide adenylyltransferase 2	Nicotinate and nicotinamide metabolism
*NTRK2*	neurotrophic tyrosine kinase, receptor, type 2	MAPK signaling pathway
*RHCG*	Rh family, C glycoprotein	
*SERPINB13*	serpin peptidase inhibitor, clade B (ovalbumin), member 13	
*SOX2*	SRY (sex determining region Y)-box 2	
*SPINK1*	serine peptidase inhibitor, Kazal type 1	
*SPRR1A*	small proline-rich protein 1A	
*TJP3*	tight junction protein 3 (zona occludens 3)	Tight junction
*TOX3*	TOX high mobility group box family member 3	
*VSNL1*	visinin-like 1	

The performance of 10-fold cross validation achieves 98.5% accuracy. We further test the classification accuracy of the network on seven independent study populations with Gene Expression Omnibus accession numbers GSE10072, GSE7670, GSE12667, GSE4824, GSE2109, GSE4573, and GSE6253, for a total of 422 samples, 232 AC and 190 SCC, from subjects of Caucasian, Asian and African descent representing 84.6%, 6.9%, and 2.8% of the data, respectively. On these independent samples, our transcriptional network classifier achieves an accuracy of 95.2%. 

The 25-gene signature identified by the classifier is unique to discriminate AC and SCC with high accuracy. Furthermore, most of these genes have been reported their specificity to lung cancer. *ABCC3*, *CLDN3*, *DPP4*, *MUC3B*, *MUC5B*, *NTRK2*, *SPINK1*, *TJP3* are specific markers of lung AC [23-29]. *KRT6A*, *KRT6B*, *KRT6C*, *KRT17*, *RHCG*, *SPRR1A*, and *VSNL1* are unique to lung SCC [30-33]. *BICD2*, *CDA*, *NMNAT2*, *SERPINB13*, and *TOX3* have no specificity to either AC or SCC but to lung cancer [34-38].

### Diagnosis of thoracic aortic aneurysm

**Figure 3:**
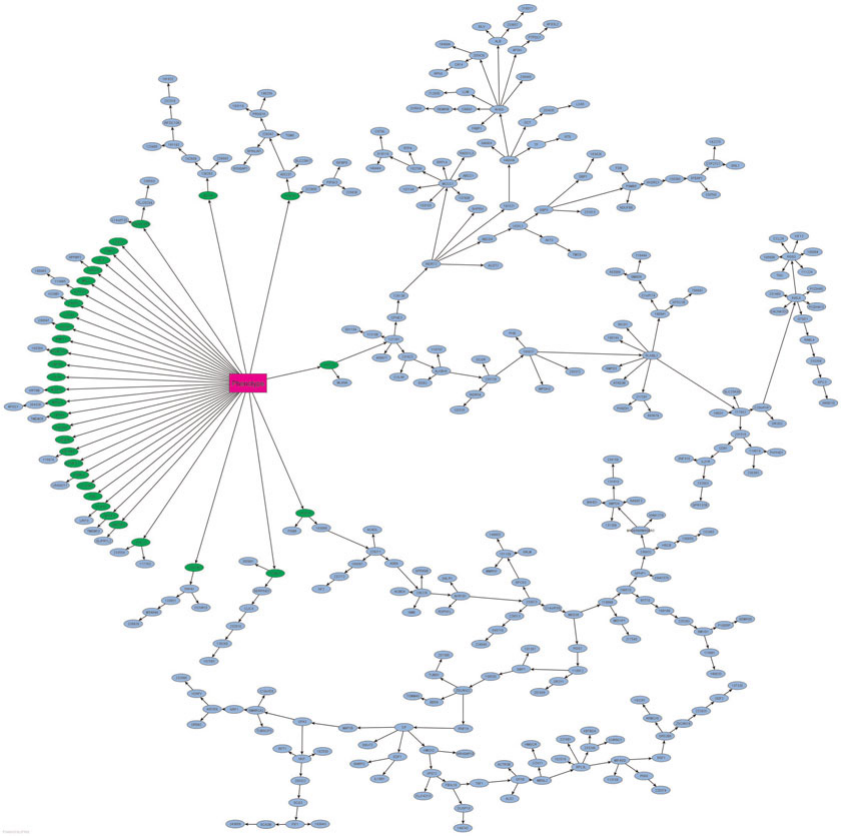
**The functional dependence network for TAA diagnosis.** There are 346 genes selected to reconstruct this network, because of their distinct expression patterns between TAA and normal samples. The signature consists of the 34 genes (in green) under the phenotypeâ€™s Markov blanket.

Thoracic aortic aneurysm (TAA) is usually asymptomatic and associated with high mortality. Identification of at-risk individuals is a challenging task. Gene expression patterns in peripheral blood cells are expected to assist the diagnosis of TAA. The data used to derive the classifier is publicly available on Gene Expression Omnibus with accession number GSE9106 [39], which involves 36 cases and 25 controls for training purpose. Peripheral blood samples were collected at Yale-New Haven Hospital. Gene expression experiments were carried out by Applied Biosystems Human Genome Survey Microarray v2.0, which is equipped with 32,878 probes. The utilization of Bayes factor in our algorithm first filters out 346 genes that are dependent on the phenotype. Bayesian network learning results in the functional dependence network shown in Figure 3. There are 34 genes under the phenotype's Markov blanket, and they form a signature for TAA diagnosis. Table 2 summarizes the annotations of the signature, where the nameless genes are provided with their probe identifies only. The genes *ABCG4*, *ARNT2*, *BCOR*, *CABP2*, *CSTF2*, *DNTTIP1*, *FGG*, *IGF2BP1*, *MAL2*, *MMP11*, *RBM16*, *TM4SF1*, *ZBTB4*, *ZNF394* are involved in connective tissue disorders and inflammatory disease, which are prerequisite to TAA. The 10-fold cross validation of the classifier yields 97% accuracy. We further examine the classifier on the independent samples, 24 cases and 9 controls, also included in the Yale data GSE9106. The accuracy on these independent samples achieves 82%, demonstrating good performance of our approach. 

**Table 2:** The signature of 34 genes for diagnosing TAA.

**Gene symbol**	**Gene title**	**Pathway**
*ABCG4*	ATP-binding cassette, sub-family G (WHITE), member 4	
*ARNT2*	aryl-hydrocarbon receptor nuclear translocator 2	
*BCOR*	BCL6 co-repressor	
*C17ORF63*	chromosome 17 open reading frame 63	
*CABP2*	calcium binding protein 2	
*CSTF2*	cleavage stimulation factor, 3' pre-RNA, subunit 2, 64kDa	
*DEFB105A*	defensin, beta 1	
*DNTTIP1*	deoxynucleotidyltransferase, terminal, interacting protein 1	
*FAF2*	Fas associated factor family member 2	
*FGG*	fibrinogen gamma chain	Coagulation system
*IGF2BP1*	insulin-like growth factor 2 mRNA binding protein 1	
*IWS1*	IWS1 homolog (S. cerevisiae)	
*KRTAP17-1*	keratin associated protein 17-1	
*KRTAP23-1*	keratin associated protein 23-1	
*MAL2*	mal, T-cell differentiation protein 2	
*MMP11*	matrix metallopeptidase 11 (stromelysin 3)	
*RBM16*	RNA binding motif protein 16	
TM4SF1	transmembrane 4 L six family member 1	
*ZBTB4*	zinc finger and BTB domain containing 4	
*ZBTB9*	zinc finger and BTB domain containing 9	
*ZNF394*	zinc finger protein 394	
*[224346]*		
*[101505]*		
*[235845]*		
*[699092]*		
*[684137]*		
*[104523]*		
*[109173]*		
*[152832]*		
*[230015]*		
*[140170]*		
*[234336]*		
*[143814]*		
*[150467]*		

### Comparisons with other methods

We contrast our proposed system biology approach with other popular algorithms that do not take into account regulatory gene interactions:

1) Principal Component Analysis with Linear Discriminant Analysis (PCA/LDA): The PCA/LDA method begins with reducing the number of genes to a small number of principal genes and then searches for a discriminative linear function on expression values to separate tissues.

2) Prediction Analysis for Microarray (PAM) [40]: PAM utilizes signal to noise ratios to pick up a signature and uses the ratios to determine the tissue types of testing samples.

3) Weighted Voting [1]: This method ranks genes by the fold change of the means of the expression values. The classification is determined by how close to the high rank genes the testing data is.

Unlike our approach, the above methods neglect dependencies among genes, so they yield worse performance than our TNC. Table 3 and Table 4 summarize the comparisons of our approach with these methods on the lung cancer and TAA studies, respectively. The results show that our approach is superior to other algorithms. On the other hand, our approach leads to more compact signatures because collinearity elimination is addressed after gene selection. The differences between our approach and other schemes are statistically significant (p<0.005), except that weighted voting performs close to ours in the lung cancer study. Although weighted voting reaches high classification accuracy on the lung cancer data, it requires a large number of genes in the signature, giving rise to overfitting problem.

**Table 3:** Performance comparisons with other methods on the lung cancer data.

**Classifier**	**Number of signature genes**	**Accuracy in independent samples**	***p*-value**
Transcriptional Network Classifier (this research)	25	95.2%	---
Principal Component Analysis with Linear Discriminant Analysis	13	91.2%	0.0047
Prediction Analysis for Microarray [40]	77	91.0%	0.0014
Weighted Voting [1]	800	93.4%	0.6240

**Table 4:** Performance comparisons with other methods on the TAA data.

**Classifier**	**Number of signature genes**	**Accuracy in independent samples**	***p*-value**
Transcriptional Network Classifier (this research)	34	81.8%	---
Principal Component Analysis with Linear Discriminant Analysis	49	71.6%	10^-7^
Prediction Analysis for Microarray [40]	41	78.4%	0.0091
Weighted Voting [1]	126	51.9%	10^-20^

## Discussion

The clinical application confirms improved accuracy of our proposed system biology approach. Literature survey on the functions of the signature genes also validates the capability of our approach to extract biologically reasonable signatures. Furthermore, the large-scale independent test on seven cohorts in the lung cancer study shows robustness of our classifier across platforms and populations. The two studies also demonstrate the capability of our method to analyze data assayed by microarrays manufactured by different makers. 

Unlike existing methods that require the operator to specify a cutoff of statistical measures to select high ranked genes, our method is threshold free for signature selection, because the signature genes are determined once the transcriptional network is modelled. For phenotype classification, we need to keep the network merely composing of the signature genes, and the remaining network can be discarded; this way can save storage resources in clinical usage. Another feature of our transcriptional network classifier is its visualization of molecular dependence network, which will provide biologists a clue for gene causality investigation.

A recent work proposes to use prior knowledge of known pathway information to select gene subnetworks as features for tissue classification [5]. However, this method will discard a major portion of the data, because a large number of genes have not been discovered their functional pathways. Dissimilar to this method, our approach fully utilizes the entire data to screen the function-dependent genes and to reconstruct the network. 

## Conclusions 

This paper uses a system biology approach to develop transcriptional network classifiers. The classifier can be thought of as a gene network perturbed by the presence of the phenotypic traits. We adopt Bayesian network framework to model the classifier. The algorithm uses Bayes factor for gene filtering, followed by collinearity elimination via network learning. The clinical applications of our approach to lung cancer subtypes classification and TAA diagnosis demonstrate high classification accuracy of the network based classifiers. The biological validation of the signatures further confirms the ability of the transcriptional network classifier to extract meaningful signatures. 

## Methods

Let *Y*_1_,*Y*_2_,...,*Y_N_* be Gaussian random variables representing the expression levels of genes 1,...,*N*, and *C* be a multinomial random variable indicating tissue conditions. We use uppercase to denote random variables and lowercase to denote their values. Our algorithm first uses Bayes factor to filter function-dependent genes and then exploit Bayesian network learning to eliminate collinearity among these selected genes. 

### Gene filtering by Bayes factor

The genes functionally dependent on the phenotype are filtered in the beginning. The filtering can be realized by Bayes factor, which evaluates for each gene the ratio of its likelihood of being dependent on the phenotype to its likelihood of being independent of the phenotype. When the Bayes factor is greater than one, the gene is selected because it is more likely to be dependent on than to be independent of the phenotype. 

### Collinearity elimination via network learning

Without loss of generality, we assume that the first *G* out of *N* genes were selected by the preceding step. The gene expression data under consideration now is *D* = {*y*_1_,*y*_2_,...,*Y_G_*,*c*}. When a gene *Y_i_* is collinearly expressed with another gene *Y_j_*, the dependence of gene *Y_i_* on the phenotype is mediated by gene *Y_j_*. In other words, our goal is to search which gene modulates gene *Y_i_* with the highest likelihood. When we find out for every gene its best upstream variable, the network is achieved. In the framework of Bayesian network, our objective is to learn from a set of candidate network models  the optimal network  fit best to the data *D*. Equivalently, we look for the highest posterior probability . Applying Bayes' theorem to  results in , where  is the prior probability of model  and  is the marginal likelihood. The computation of  is to average out  from the likelihood function , where  is the values of the random vector  parameterizing the distribution of *y*_1_,*y*_2_,...,*Y_G_*,*C* conditional on . We can exploit the local Markov properties encoded by the network  to rewrite the joint probability  as

,

where *pa*(*x*) denotes the values of the parents *Pa*(*X*) of random variables *X*, and  is the subset of parameters used to describe the dependence of variable *X* on its parents.

In this paper, we model a gene *Y_g_* to be dependent on either the phenotype *C* or another single gene*Y_a_*, and the phenotype *C* is the root in the network without parents. We further can assume the *J* samples in the database are independent. The likelihood function becomes

,

where the subscripts *j* indicate the *j*th sample. The first term can be estimated by sample frequencies, and the second term can be derived using linear Gaussian model [41]. The marginal likelihood function is the solution of the integral

,

Due to limited space, we in this paper do not present the detailed computation, which can be derived from [41]. Finally, the determination of the best Bayesian network model is .

### Sample classification

The phenotype classification  of a sample is to find the maximum probability of the tissue class that the sample belongs to, conditional on the expression values of the sample. The formulation for the classification is as follows:

,

The application of Bayes' theorem leads to 



,

where the second equality holds because the denominator *p*(*y*_1_,*y*_2_,...,*y*_G_) in the first line is not a function of *c*. Since only genes directly dependent on the phenotype variable *C* matter in the maximization, the tissue classification becomes

,

where *H* denotes the set of genes that are the children of the phenotype *C* in the network and assemble a signature. Equivalently, the set *H* of genes corresponds to the genes under the phenotype's Markov blanket.

## Competing interests

The authors declare that they have no competing interest.

## Authors' contributions

HHC designed the method and conducted the analysis; MFR directed the study; both authors prepared the manuscript.
